# Downregulation of PACAP and the PAC1 Receptor in the Basal Ganglia, Substantia Nigra and Centrally Projecting Edinger–Westphal Nucleus in the Rotenone model of Parkinson’s Disease

**DOI:** 10.3390/ijms241411843

**Published:** 2023-07-24

**Authors:** Máté Fehér, Zsombor Márton, Ákos Szabó, János Kocsa, Viktória Kormos, Ágnes Hunyady, László Ákos Kovács, Balázs Ujvári, Gergely Berta, József Farkas, Nóra Füredi, Tamás Gaszner, Bence Pytel, Dóra Reglődi, Balázs Gaszner

**Affiliations:** 1 Department of Anatomy, Medical School, University of Pécs, Szigeti út 12, H-7624 Pécs, Hungary; fehermat@gmail.com (M.F.); martonzsomi@gmail.com (Z.M.); akos.szabo07@gmail.com (Á.S.); kocsajano43@gmail.com (J.K.); laszlo.akos.kovacs@aok.pte.hu (L.Á.K.); balazs.ujvari@aok.pte.hu (B.U.); jozsef.farkas@aok.pte.hu (J.F.); furedinora@gmail.com (N.F.); gaszner.tamas@gmail.com (T.G.); bencepytel@gmail.com (B.P.); dora.reglodi@aok.pte.hu (D.R.); 2 Research Group for Mood Disorders, Centre for Neuroscience, University Medical School, University of Pécs, Szigeti út 12, H-7624 Pécs, Hungary; 3 Department of Neurosurgery, Kaposi Mór Teaching Hospital, Tallián Gy. u. 20-32, H-7400 Kaposvár, Hungary; 4 Department of Pharmacology and Pharmacotherapy, Medical School, University of Pécs, Szigeti út 12, H-7624 Pécs, Hungary; viktoria.kormos@aok.pte.hu (V.K.); agnes.hunyady@gmail.com (Á.H.); 5 Department of Medical Biology and Central Electron Microscopic Laboratory, Medical School, University of Pécs, Szigeti út 12, H-7624 Pécs, Hungary; gergely.berta@aok.pte.hu; 6 ELKH-PTE PACAP Research Group, Department of Anatomy, Medical School, University of Pécs, Szigeti út 12, H-7624 Pécs, Hungary

**Keywords:** caudate-putamen, globus pallidus, entopeduncular nucleus, cortex, rotarod, non-motor symptoms of Parkinson’s disease, rat

## Abstract

Numerous in vitro and in vivo models of Parkinson’s disease (PD) demonstrate that pituitary adenylate cyclase-activating polypeptide (PACAP) conveys its strong neuroprotective actions mainly via its specific PAC1 receptor (PAC1R) in models of PD. We recently described the decrease in PAC1R protein content in the basal ganglia of macaques in the 1-methyl-4-phenyl-1,2,3,6-tetrahydropyridine (MPTP) model of PD that was partially reversed by levodopa therapy. In this work, we tested whether these observations occur also in the rotenone model of PD in the rat. The rotarod test revealed motor skill deterioration upon rotenone administration, which was reversed by benserazide/levodopa (B/L) treatment. The sucrose preference test suggested increased depression level while the open field test showed increased anxiety in rats rendered parkinsonian, regardless of the received B/L therapy. Reduced dopaminergic cell count in the substantia nigra pars compacta (SNpc) diminished the dopaminergic fiber density in the caudate-putamen (CPu) and decreased the peptidergic cell count in the centrally projecting Edinger–Westphal nucleus (EWcp), supporting the efficacy of rotenone treatment. RNAscope in situ hybridization revealed decreased PACAP mRNA (*Adcyap1*) and PAC1R mRNA (*Adcyap1r1*) expression in the CPu, globus pallidus, dopaminergic SNpc and peptidergic EWcp of rotenone-treated rats, but no remarkable downregulation occurred in the insular cortex. In the entopeduncular nucleus, only the *Adcyap1r1* mRNA was downregulated in parkinsonian animals. B/L therapy attenuated the downregulation of *Adcyap1* in the CPu only. Our current results further support the evolutionarily conserved role of the PACAP/PAC1R system in neuroprotection and its recruitment in the development/progression of neurodegenerative states such as PD.

## 1. Introduction

Pituitary adenylate cyclase-activating polypeptide (PACAP) is a phylogenetically conserved neuropeptide with strong neuroprotective effects from invertebrates to lower vertebrates and humans [1,2,3,4,5,6,7,8,9]. PACAP is the ligand of three G-protein-coupled receptors. VPAC1 and VPAC2 receptors bind both PACAP and vasoactive intestinal polypeptide (VIP). In contrast, the PAC1 receptor (PAC1R) binds PACAP, but not VIP, with high affinity, and the neuroprotective action of PACAP is attributed to its signaling via PAC1R [8,10].

PACAP and PAC1Rs were found to be expressed in numerous organ systems [10] including a wide range of brain areas. The occurrence of PACAP and PAC1R in the basal ganglia and substantia nigra of multiple species suggested their phylogenetically conserved role in the neuroprotection of these centers [11,12,13,14]. Dopaminergic neuron loss in the substantia nigra pars compacta (SNpc) and consequent dopamine depletion in the striatum is a well-known neuropathology that contributes to the development of motor symptoms of Parkinson’s disease (PD) [15]. Much less is known about the background of mood-related non-motor symptoms of PD, such as depression [16]. Recently, we found that the centrally projecting Edinger–Westphal nucleus (EWcp) suffers neurodegeneration in the rotenone model of PD which contributes to depressed mood [17]. Importantly, these peptidergic neurons were shown to co-express PACAP (*Adcyap1*) mRNA [18], and we saw their recruitment in various models for depression and anxiety applied in mice lacking one or both functional *Adcyap1* gene alleles [19,20,21,22].

The beneficial role of PACAP/PAC1R signaling in various models of neurodegenerative states including PD has been proven [6,23,24]. For example, toxicity studies on dopaminergic cell cultures revealed that the deleterious effect of 6-hydroxydopamine (6-OHDA) [25], salsolinol [26]; 1-methyl-4-phenyl-1,2,3,6-tetrahydropyridine (MPTP) [27,28], or rotenone [29,30,31] may be reversed by PACAP treatment (for a review see [8,23]). In vivo experiments further supported the evolutionarily conserved neuroprotective action of PACAP in various models of PD. For instance, we showed in snails that the rotenone-induced [32], and in rats the 6-OHDA-evoked Parkinson-like state can be reversed by PACAP treatment [32,33,34,35]. In line with this, PACAP administration in mice rendered parkinsonian by MPTP treatment also exerted a beneficial effect [28,36,37]. The role of endogenous PACAP in neuroprotection was also shown in knockout mice [38,39]. More recently, we examined the basal ganglia of macaques and found that the PAC1R protein content is markedly reduced in an MPTP-induced parkinsonism-like state, which was partially reversed by benserazide/levodopa (B/L) treatment [14]; however, in this study we did not examine whether the PACAP mRNA (*Adcyap1*) was affected.

Human studies also support the reduced blood PACAP level associated with motor [40] and non-motor [41] symptoms of PD. In contrast, in the cerebrospinal fluid samples of subjects with PD, no alteration of the PACAP level was detected [42], suggesting that, in some brain areas, changes at the PAC1R may also have a significant role.

In order to more deeply understand the role of PACAP and PAC1R in PD, we aimed to test the effect of the rotenone-induced parkinsonism-like state and B/L therapy in the rat. We hypothesized that the motor deficit and the dopaminergic neuron loss in the substantia nigra pars compacta (SNpc) is associated with decreased *Adcyap1* and *Adcyap1r1* mRNA expression; moreover, with reduced PAC1R content in the caudate putamen (CPu), globus pallidus (GP), entopeduncular nucleus (EP, the rodent equivalent of the primate globus pallidus internus) and SNpc. We also hypothesized that altered *Adcyap1* and *Adcyap1r1*/PAC1R content of the EWcp may be associated with increased anxiety and a depression-like state.

To test these hypotheses, rats were rendered parkinsonian by chronic subcutaneous (sc) rotenone administration and a subgroup was treated with B/L. Rotarod performance test (RPT) was used to assess the rats’ motor skills, while the mood status was evaluated with sucrose preference (SPT) and open field tests (OFT). RNAscope in situ hybridization (ISH) combined with immunofluorescence was used to semi-quantify the *Adcyap1* and *Adcyap1r1* mRNA and PAC1R protein content.

## 2. Results

### 2.1. The Validity of Our Model

**Figure 1:**
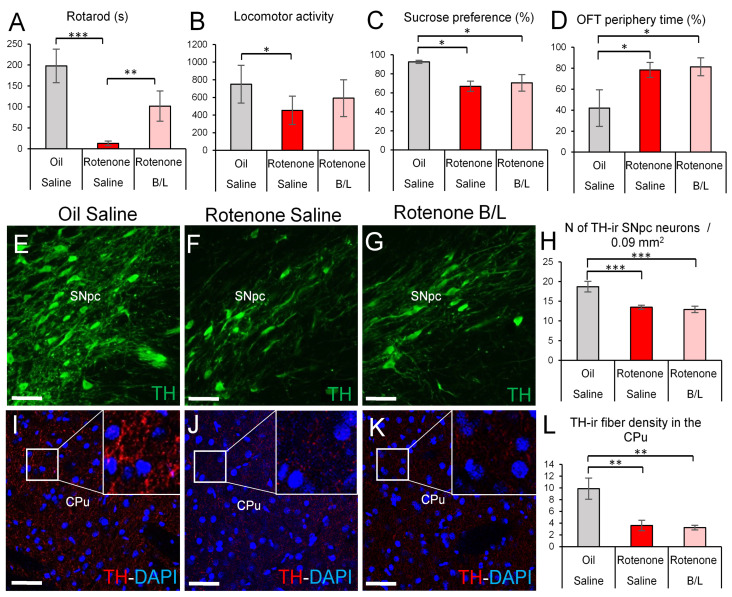
Behavioral and morphological tests for efficacy of the rotenone model. Rats treated with rotenone (red bars) spent less time on the rotarod device (**A**) and showed decreased locomotor activity (**B**) in the open field test (OFT) than oil- and saline-injected controls (gray bars). Reduced sucrose preference (**C**) suggested increased anhedonia in rotenone-treated rats, and they spent a longer period of time next to the walls (**D**) of the open field device, suggesting increased anxiety. Benserazide/levodopa (B/L) administration in rotenone-treated rats (pink bars) improved the motor performance in the rotarod test but remained ineffective on the mood state. Tyrosine hydroxylase (TH) immunolabeling (green in (**E**–**G**)) in the substantia nigra pars compacta (SNpc) revealed decreased dopaminergic cell counts (**H**) in rotenone-treated animals. Reduced density of TH immunoreactivity (ir) (red in (**I**–**K**)) was observed in the neuropil of the caudate-putamen (CPu) upon rotenone treatment (**L**). Blue: 4′,6-diamidino-2-phenylindole (DAPI) nuclear counterstaining. N = 8–12 in (**A**–**D**), N = 6–8 in (**E**–**L**). * *p* < 0.05, ** *p* < 0.01, *** *p* < 0.001, according to Tukey’s post-hoc test. Bars: 50 µm.

Rotenone exposure resulted in a drastic deterioration (ANOVA: main effect of treatment F2,17 = 9.06; *p* < 0.01) of motor performance (Figure 1A). Control rats spent 197.30 ± 39.99 s on the rotating rod. Rats rendered parkinsonian spent much shorter (13.0 ± 5.3 s; *p* < 0.0001) time on the device. B/L therapy improved the motor performance of rotenone-treated rats (101.85 ± 5.3 s, *p* < 0.001) that did not differ from controls significantly (*p* = 0.10). Rotenone-treated (ANOVA: F2,17 = 4.29; *p* = 0.027) rats showed reduced open field locomotor activity (*p* = 0.043, Figure 1B) compared to controls, while B/L-injected parkinsonian animals did not differ from controls (*p* = 0.26).

Rotenone injections also affected the depression level (ANOVA: F2,26 = 4.21; *p* = 0.025). Parkinsonian rats showed an increased level of anhedonia compared to controls (*p* < 0.01) as they showed reduced sucrose preference (Figure 1C), regardless of whether they received anti-Parkinson medication (*p* = 0.033). In line with this, ANOVA revealed the main effect of treatment on the time spent in the periphery of the open field box as significant (F2,26 = 4.21; *p* = 0.025). Rotenone-injected rats showed increased anxiety (*p* = 0.05) that was not affected by B/L injections (*p* = 0.96), as they also spent longer periods of time along the walls and in the corners (Figure 1D).

Dopaminergic neuron loss in the SNpc is the most important histopathological indicator of PD. In line with this, the number of TH-immunoreactive (ir) neurons was affected by the treatment (ANOVA: F2,21 = 11.83; *p* < 0.001). The number of dopaminergic neurons was reduced by 28% in rotenone-injected rats, compared to controls (*p* < 0.001) (Figure 1E–H), and the B/L medication did not affect the number of TH-ir cells in the SNpc (*p* = 0.60). In accordance with this (Figure 1I–L, ANOVA: F2,19 = 9.61; *p* = 0.001), the dopaminergic fiber density was reduced in the CPu in both rotenone-treated groups regardless (*p* = 0.001) of the anti-Parkinson medication (*p* = 0.001) compared to the oil-injected control rats. In line with the SNpc, we also detected reduced peptidergic cell count in the EWcp (see also in Section 2.2.5) (ANOVA: F2,21 = 22.24; *p* < 10^−5^) in rotenone-treated animals (*p* < 10^−4^). B/L treatment of parkinsonian rats did not reverse the neuron loss in the EWcp either (*p* = 0.25).

### 2.2. Morphological Results

To test whether *Adcyap1* and *Adcyap1r1* mRNAs, moreover the PAC1R protein content of the brain is affected in the rotenone model of PD, RNAscope ISH and immunofluorescent labeling were carried out in the CPu, GP, EP, SN and EWcp. In order to test whether the anticipated alterations are restricted to the PD-affected regions, we also assessed the insular cortex because it is either not affected by PD, or it develops neurodegenerative changes in the late phase of the disease [43].

#### 2.2.1. Caudate-Putamen

**Figure 2:**
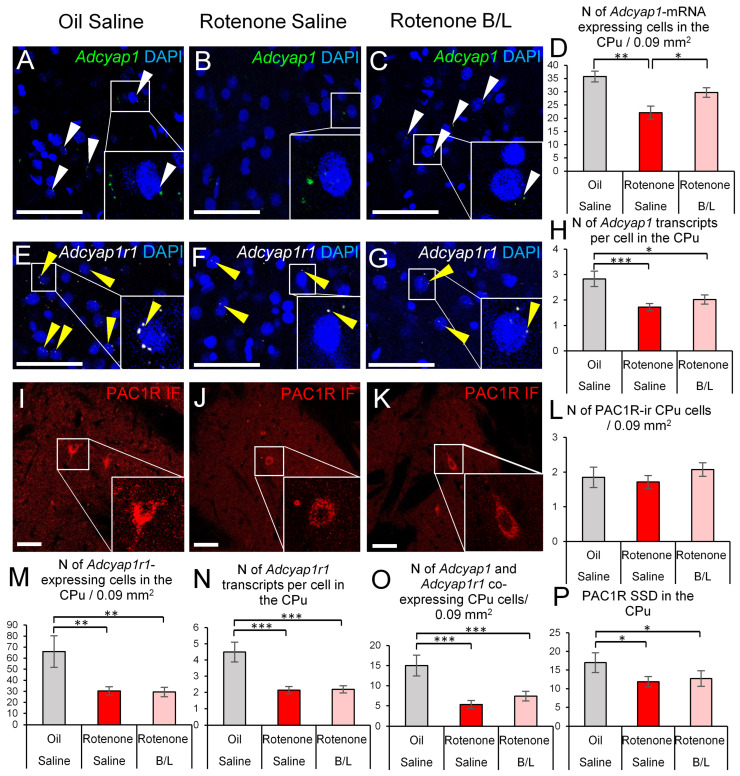
*Adcyap1* and *Adcyap1r1* mRNA expression, and PAC1R immunoreactivity in the caudate-putamen (CPu). Representative images show *Adcyap1* mRNA transcripts (green in (**A**–**C**), white arrowheads), *Adcyap1r1* mRNA signal puncta (white, (**E**–**G**), yellow arrowheads) and 4′,6-diamidino-2-phenylindole (DAPI) nuclear counterstaining (blue). Some cells are shown in higher magnification inserts within the same panels. Panels (**I**–**K**) show PAC1R-immunoreactive (ir) somata (red) in the CPu. Histogram (**D**) shows the number of *Adcyap1*-expressing cells. Panel (**H**) illustrates the count of *Adcyap1* transcripts per cell. Histogram (**L**) shows the number of PAC1R-ir cell counts. Histogram (**M**) shows the count of cells that contained *Adcyap1r1.* Panel (**N**) describes the magnitude of *Adcyap1r1* expression. The count of *Adcyap1* and *Adcyap1r1* co-expressing cells is shown in histogram (**O**). The specific signal density (SSD) of PAC1R protein is shown in panel (**P**). Gray bars: oil- and saline-injected controls; red bars: rats treated with rotenone; pink bars: benserazide/levodopa (B/L)-treated parkinsonian rats. N = 6–8 * *p* < 0.05, ** *p* < 0.01, *** *p* < 0.001, according to Tukey’s post-hoc test. Bars: 50 µm.

ANOVA found the main effect of the treatment significant (F2,21 = 8.25; *p* < 0.01) on *Adcyap1* expression. Rotenone treatment reduced the number of *Adcyap1*-expressing cells by 38% (*p* < 0.001) in the CPu; that effect was reversed by B/L treatment (*p* = 0.017, Figure 2A–D). When comparing the magnitude of *Adcyap1* expression in the CPu cells (Figure 2H), it appeared that the rotenone treatment (F2,21 = 7.34; *p* = 0.003) reduced the expression (*p* = 0.002) and B/L treatment did not restore (*p* = 0.23) the normal count of *Adcyap1* transcripts per cell.

The count of *Adcyap1r1*-expressing cells was also affected (F2,21 = 5.33; *p* = 0.01) and the cell count was also reduced (Figure 2E–G,M) in parkinsonian rats (*p* = 0.008), which was not prevented by B/L administration (*p* = 0.72). In line with this, the number of *Adcyap1r1* transcripts was also reduced by rotenone exposure (ANOVA: F2,21 = 15.45; *p* < 0.0001) both in saline- (*p* < 0.001) and B/L-injected (*p* < 0.001) groups (Figure 2N). We also counted the number of *Adcyap1r1*-expressing cells which showed similar sensitivity to rotenone (ANOVA: F2,21 = 10.34; *p* < 0.001) and its effect (*p* < 0.001) was not reversed (Figure 2O) by the anti-Parkinson medication (*p* = 0.49). In our PAC1R immunolabeling, we recognized only a few PAC1R-ir perikarya in the CPu (Figure 2I–L) and their number was not affected by rotenone treatment (ANOVA: F2,21 = 0.80; *p* = 0.46). Contrary to this, but in line with the *Adcyap1r1* mRNA pattern, the PAC1R SSD (ANOVA: F2,21 = 4.82; *p* = 0.022) was decreased (Figure 2P) by rotenone treatment (*p* = 0.046), and which was not reversed by the B/L medication (*p* = 0.95) in the CPu.

#### 2.2.2. Globus Pallidus

**Figure 3:**
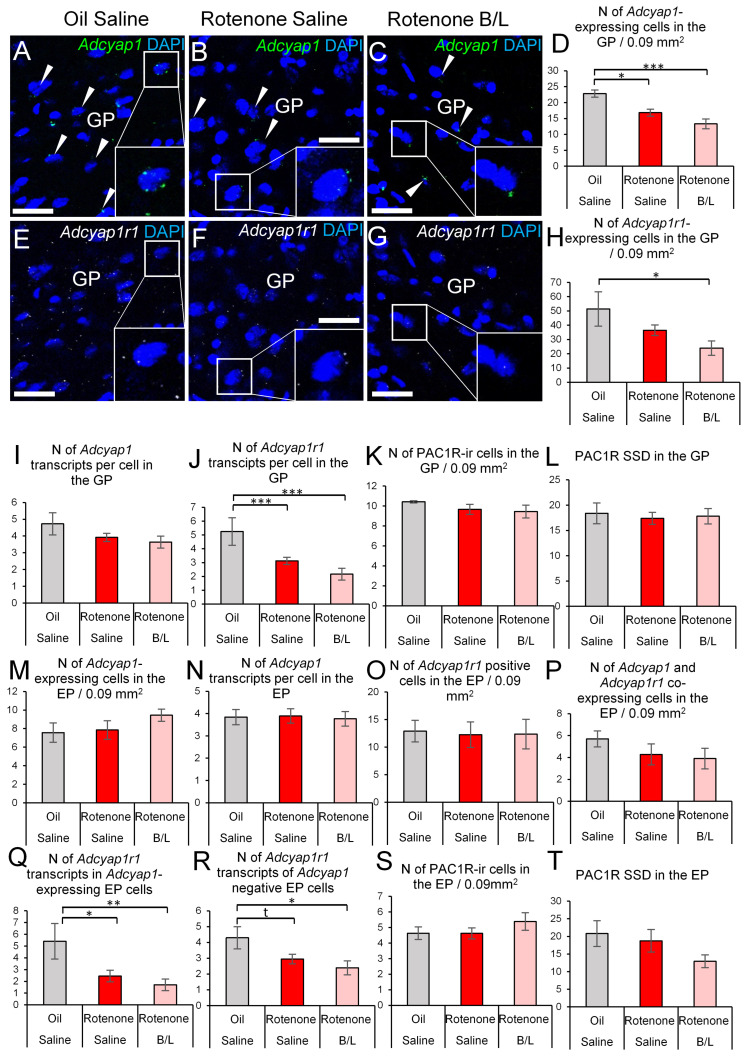
*Adcyap1* and *Adcyap1r1* mRNA expression in the globus pallidus (GP) and entopeduncular nucleus (EP). Representative images show *Adcyap1* mRNA transcripts (green in (**A**–**C**), white arrowheads) and *Adcyap1r1* mRNA signal puncta (white, (**E**–**G**)) counterstained with 4′,6-diamidino-2-phenylindole (DAPI, blue) in the GP. Some cells were shown in higher magnification insets within the same panels. Histogram (**D**) shows the number of *Adcyap1*-expressing cells in the GP. Panel (**H**) shows the count of cells that contained *Adcyap1r1* in the GP. Histogram (**I**) illustrates the count of *Adcyap1* transcripts per GP cell. Panel (**J**) describes the magnitude of *Adcyap1r1* expression in the cells of GP. The count of PAC1R-immunoreactive (ir) cells in the GP is shown in (**K**). The specific signal density (SSD) of PAC1R protein in the GP is shown in panel (**L**). Histogram (**M**) shows the number of *Adcyap1*-expressing cells in the EP. Histogram (**N**) illustrates the count of *Adcyap1* transcripts per EP cell. Histogram (**O**) shows the number of *Adcyap1r1*-expressing cells in the EP. The count of *Adcyap1* and *Adcyap1r1* co-expressing cells in the EP is shown in histogram (**P**). Panel (**Q**) shows the number of *Adcyap1r1* transcripts in *Adcyap1*-expressing EP cells. Histogram (**R**) indicates the number of *Adcyap1r1* transcripts in *Adcyap1*-negative EP cells. The count of PAC1R-ir cells in the EP is shown in (**S**). The SSD of PAC1R protein in the EP is shown in panel (**T**). Gray bars: oil- and saline-injected controls; red bars: rats treated with rotenone; pink bars: benserazide/levodopa (B/L)-treated parkinsonian rats. N = 6–8. * *p* < 0.05, ** *p* < 0.01, *** *p* < 0.001, t: trend (*p* = 0.06), according to Tukey’s post-hoc test. Bars: 30 µm.

The *Adcyap1* expression was affected by the treatment (ANOVA: F2,17 = 10.84; *p* < 0.001) and the rotenone reduced the number of *Adcyap1*-expressing cells (*p* = 0.027), while B/L treatment did not reverse this effect (*p* = 0.17, Figure 3A–D). In contrast, the number of *Adcyap1* signal puncta per cell was not significantly reduced in the model (ANOVA: F2,17 = 1.76; *p* = 0.19, Figure 3I).

Although the count of *Adcyap1r1*-expressing cells was affected in the model, (ANOVA: F2,17 = 4.29; *p* = 0.027; Figure 3E–H) the post-hoc test did not confirm that the rotenone reduced the number of cells that contained *Adcyap1r1* signal puncta (*p* = 0.26). Counting of *Adcyap1r1* mRNA dots revealed that the treatment influenced the expression (ANOVA: F2,19 = 8.20; *p* = 0.002), that was reduced by rotenone exposure (*p* = 0.009), regardless whether rats received B/L injections (*p* = 0.14, Figure 3J). Neither the number of PAC1R immunopositive cells (ANOVA: F2,19 = 0.66; *p* = 0.52 Figure 3K) nor the PAC1R SSD (ANOVA: F2,19 = 0.06; *p* = 0.91, Figure 3L) was influenced by the treatment.

#### 2.2.3. Entopeduncular Nucleus

The *Adcyap1* expression was not affected in the EP by the treatment as neither the *Adcyap1*-containing cell count (ANOVA: F2,21 = 1.19; *p* = 0.32, Figure 3M) nor the number of mRNA transcripts per cell was altered (ANOVA: F2,21 = 0.06; *p* = 0.93, Figure 3N).

Neither the count of only *Adcyap1r1* mRNA positive (ANOVA: F2,21 = 1.04; *p* = 0.326, Figure 3O) nor the number of *Adcyap1r* and *Adcyap1r1* co-expressing cells (ANOVA: F2,21 = 0.85; *p* = 0.45, Figure 3P) was altered significantly. The counting of *Adcyap1r1* transcripts in *Adcyap1* mRNA-containing cells revealed that the rotenone exposure influenced (ANOVA: F2,17 = 5.72; *p* < 0.05) the expression: a downregulation (*p* = 0.01) occurred that was not reversed by the therapy (*p* = 0.42, Figure 3Q). Although a similar pattern was found in *Adcyap1* negative cells also (ANOVA: F2,17 = 3.92; *p* < 0.05), the post-hoc test found only a trend of rotenone effect (*p* = 0.06, Figure 3R). Neither the PAC1-ir cell count (ANOVA: F2,21 = 0.88; *p* = 0.42, Figure 3S), nor the SSD of PAC1 immunoreactivity (ANOVA: F2,21 = 2.14; *p* = 0.14, Figure 3T) was influenced significantly.

#### 2.2.4. Substantia Nigra, Pars Compacta

**Figure 4:**
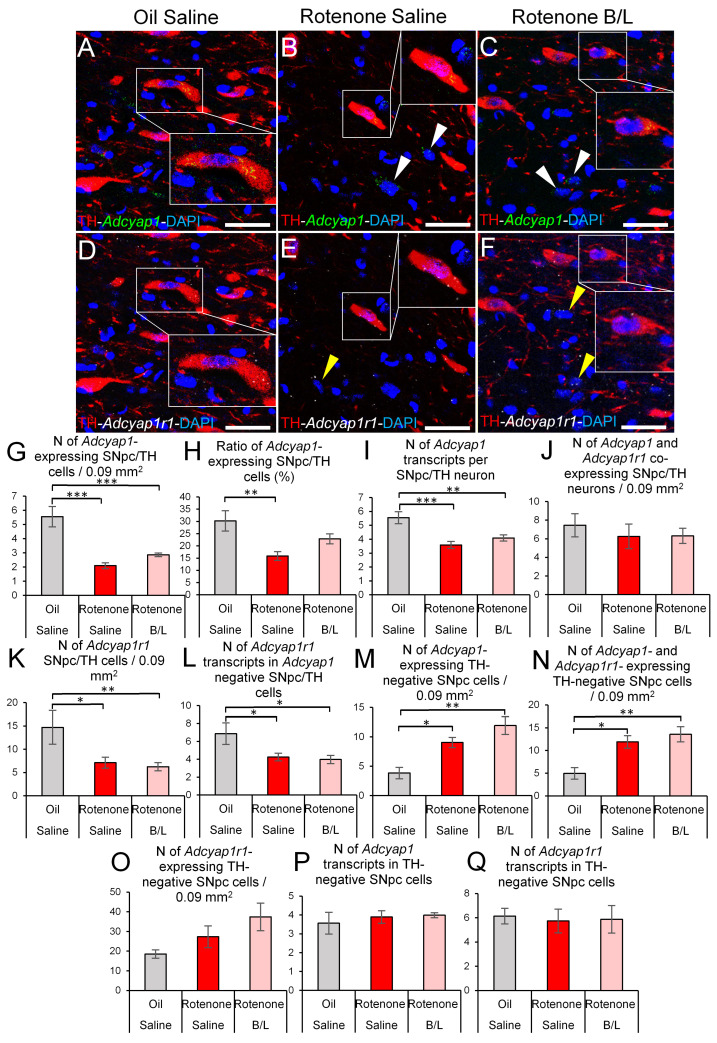
*Adcyap1* and *Adcyap1r1* mRNA expression in the substantia nigra pars compacta (SNpc). Representative images show *Adcyap1* mRNA transcripts (green in (**A**–**C**), white arrowheads) and *Adcyap1r1* mRNA signal puncta (white, (**D**–**F**)) in tyrosine hydroxylase (TH, red) immunoreactive (ir) and non-dopaminergic (white and yellow arrowheads) cells. Nuclear counterstaining: 4′,6-diamidino-2-phenylindole (DAPI, blue). Some cells are shown in higher magnification insets within the same panels. Histogram (**G**) shows the number of *Adcyap1*-expressing TH-ir cells. Panel (**H**) shows the ratio of *Adcyap1*-expressing TH-ir neurons. Histogram (**I**) illustrates the count of *Adcyap1* transcripts in dopaminergic neurons. Panel (**J**) shows the number of dopaminergic cells that co-express *Adcyap1* and *Adcyap1r1.* Graph (**K**) illustrates the count of TH-ir cells that contained *Adcyap1r1*. Histogram (**L**) indicates the number of *Adcyap1r1* transcripts in non-dopaminergic *Adcyap1*-expressing cells. Panel (**M**) shows the count of *Adcyap1*-expressing non-dopaminergic cells (see also the white arrowheads in (**A**–**C**)). Histogram (**N**) shows the count of *Adcyap1* and *Adcyap1r1* co-expressing non-dopaminergic cells (see also yellow arrowheads in (**D**–**F**)). Graph (**O**) shows the number of TH-immunonegative *Adcyap1r1*-expressing cells. The number of *Adcyap1r1* transcripts in non-dopaminergic SNpc cells is depicted in graph (**P**). Histogram (**Q**) illustrates the magnitude of *Adcyap1r1* mRNA expression in non-dopaminergic cells of the SNpc. Gray bars: oil- and saline-injected controls; red bars: rats treated with rotenone; pink bars: benserazide/levodopa (B/L)-treated parkinsonian rats. N = 6–8. * *p* < 0.05, ** *p* < 0.01, *** *p* < 0.001, according to Tukey’s post-hoc test. Bars: 25 µm.

As described above, the reduction of SNpc/TH-ir cell count confirmed the efficacy of our rotenone model. When we assessed the *Adcyap1* expression in the TH-ir neurons, we saw that the rotenone treatment affected both the count (ANOVA: F2,17 = 27.96; *p* < 10^−5^) and ratio (ANOVA: F2,17 = 8.06; *p* < 0.01) of *Adcyap1*-expressing dopaminergic neurons in the SNpc. The rotenone treatment reduced the absolute number of *Adcyap1*-expressing TH cells by 62% (Figure 4A–C,G; *p* < 10^−6^). In line with this, the proportion of *Adcyap1*-expressing TH-ir cells was lower upon rotenone treatment (Figure 4H, *p* < 0.001). The B/L treatment did not significantly reverse the effect of rotenone on the absolute count of *Adcyap1*-expressing TH cells (Figure 4G, rotenone vs. B/L: *p* = 0.06). On the other hand, the ratio of *Adcyap1*-expressing TH-ir cells in the B/L-administered rats did not differ significantly from the controls either (Figure 4H, *p* = 0.07). The rotenone treatment (ANOVA: F2,21 = 10.65; *p* < 0.001) decreased the number of *Adcyap1* mRNA transcripts (Figure 4A–C, in SNpc/TH neurons compared to oil-injected controls (Figure 4I, *p* < 0.001); that effect was not reversed by the anti-Parkinson medication (*p* = 0.36). It has to be noted that the count of SNpc TH cells that co-expressed both *Adcyap1* and *Adcyap1r1* was not affected by rotenone exposure (Figure 4J, ANOVA: F2,17 = 0.24; *p* = 0.78).

Rats rendered parkinsonian exerted approximately 50% less SNpc/TH cells that expressed *Adcyap1r1* mRNA (ANOVA: F2,21 = 4.93; *p* = 0.017) than oil-injected controls (Figure 4D–F,K; *p* = 0.012). The B/L medication did not change the count of *Adcyap1r1*-expressing TH cells. A similar pattern was observed in the dynamics of *Adcyap1r1* mRNA amount per cell also (ANOVA: F2,21 = 5.20; *p* = 0.014). The rotenone treatment halved the *Adcyap1r1* expression (Figure 4L, *p* = 0.027) and was not affected by B/L treatment (*p* = 0.93).

The assessment of non-dopaminergic (i.e., TH negative) SNpc cells revealed that the treatment affected the number of cells that express *Adcyap1* alone (ANOVA: F2,20 = 8.80; *p* = 0.001) or both *Adcyap1* and *Adcyap1r1* (ANOVA: F2,20 = 7.06; *p* < 0.01). The number of non-dopaminergic SNpc cells that expressed *Adcyap1* increased in both saline (*p* = 0.03) and B/L-injected (*p* = 0.001) parkinsonian rats (Figure 4A–C,M). We observed very similar dynamics in the number of cells that co-expressed *Adcyap1* and *Adcyap1r1* (Figure 4N, oil vs. rotenone-saline: *p* = 0.028; oil vs. rotenone-B/L: *p* = 0.004). The number of non-dopaminergic SNpc cells that express only *Adcyap1r1* (but not *Adcyap1*) (Figure 4O) remained statistically unaffected (ANOVA: F2,21 = 1.44; *p* = 0.25). When we counted the number of *Adcyap1* (Figure 4P, ANOVA: F2,21 = 0.3544; *p* = 0.70) and *Adcyap1r1* (Figure 4Q, ANOVA: F2,21 = 0.02; *p* = 0.97) transcripts in TH-negative cells, we did not detect any changes in the magnitude of mRNA expression.

**Figure 5:**
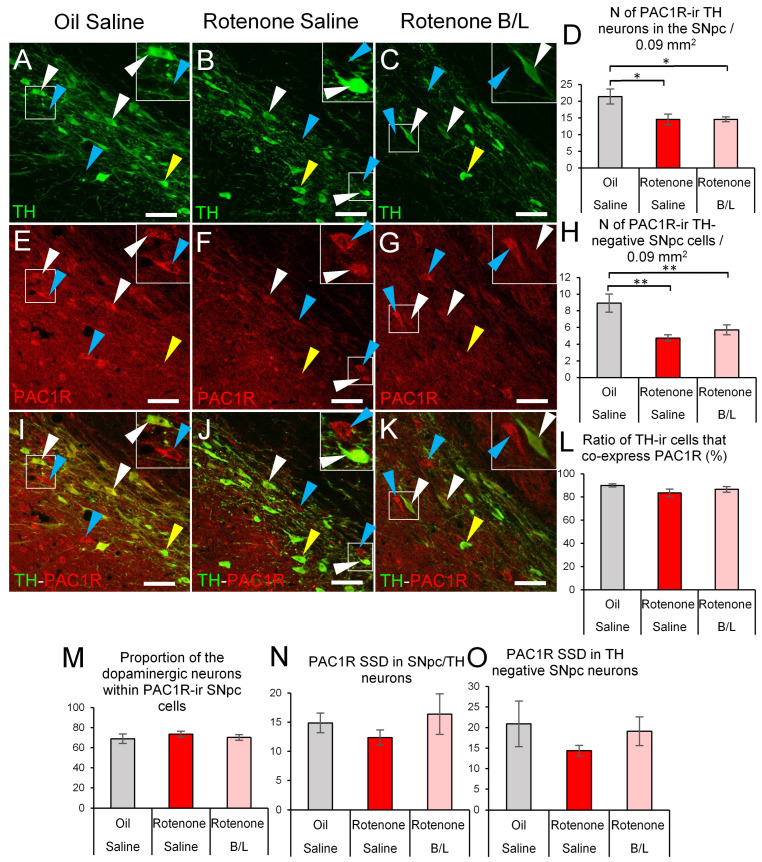
PAC1R immunoreactivity in the substantia nigra pars compacta (SNpc). Representative images of tyrosine hydroxylase (TH, green, (**A**–**C**)) and PAC1R (red, (**E**–**G**)) double labeling and their overlay (**I**–**K**). Histogram (**D**) shows the count of TH immunoreactive (ir) cells that were also PAC1R immunopositive (see also white arrowheads in the images, and insets). Panel (**H**) illustrates the number of PAC1R-ir cells that were not dopaminergic (also pointed by blue arrowheads in the confocal photomicrographs and shown in insets). Graph (**L**) illustrates that most of the dopaminergic neurons were also positive for PAC1R. Yellow arrowheads in images indicate few TH-ir cells that were immunonegative for PAC1R. Graph (**M**) shows the proportion of dopaminergic neurons in the PAC1R-ir cell population if the SNpc. Histogram (**N**) illustrates the specific signal density (SSD) of PAC1R immunosignal in dopaminergic SNpc neurons. Panel (**O**) describes the SSD of PAC1R immunosignal in non-dopaminergic cells of the SNpc. Gray bars: oil- and saline-injected controls; red bars: rats treated with rotenone; pink bars: benserazide/levodopa (B/L)-treated parkinsonian rats. N = 6–8. * *p* < 0.05, ** *p* < 0.01, according to Tukey’s post-hoc test. Bars: 50 µm.

We examined the PAC1R-TH co-existence also at the protein level in the SNpc. The absolute count of PAC1R-ir SNpc/TH cells was affected by the treatment (ANOVA: F2,20 = 6.11; *p* < 0.01). Rotenone administration reduced the PAC1R-ir SNpc/TH cell count by 32% (Figure 5D, *p* = 0.012), while the B/L administration did not affect this parameter (*p* = 0.99). We also counted the PAC1R-ir non-dopaminergic SNpc neurons, and observed that rotenone (ANOVA: F2,20 = 9.63; *p* < 0.01) reduced the number of these cells also (Figure 5H, *p* = 0.001), regardless of whether rats received B/L treatment (*p* = 0.25). To test whether the co-existence of PAC1R in dopaminergic and non-dopaminergic cells affects the neurons’ vulnerability, we determined the proportion of PAC1R-ir SNpc dopaminergic cells and observed that nearly 90% of the SNpc/TH cells were also ir for PAC1R (Figure 5I–L), and this ratio was not altered in the model (ANOVA: F2,20 = 1.06; *p* = 0.35). In line with this, when we assessed the proportion of TH-ir cells within the total PAC1R-ir cell population we saw that approximately 70% of PAC1R-ir SNpc cells were also positive for TH (Figure 5I–K,M), and we did not see any change in this proportion in our model (ANOVA: F2,20 = 0.51; *p* = 0.60). Finally, we also measured the PAC1R SSD that was altered neither in TH-ir (ANOVA: F2,20 = 0.57; *p* = 0.57, Figure 5N) nor in non-dopaminergic (ANOVA: F2,20 = 0.59; *p* = 0.56, Figure 5O).

#### 2.2.5. Centrally Projecting Edinger–Westphal Nucleus

**Figure 6:**
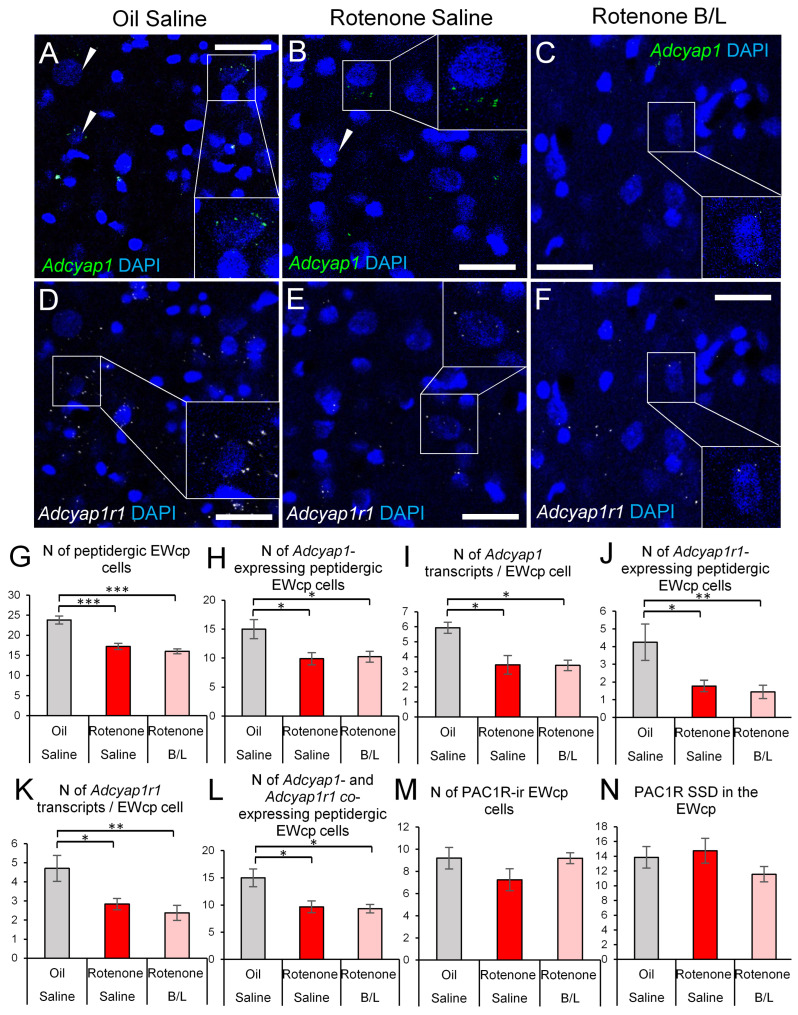
*Adcyap1* and *Adcyap1r1* mRNA expression in the centrally projecting Edinger–Westphal nucleus (EWcp). Representative images show *Adcyap1* mRNA transcripts (green in (**A**–**C**), white arrowheads) and *Adcyap1r1* mRNA signal puncta (white, (**D**–**F**)) in sections counterstained with 4′,6-diamidino-2-phenylindole (DAPI, blue). Some cells were shown in higher magnification insets within the same panels. Histogram (**G**) shows the number of peptidergic cells characterized by larger nuclei and pale, euchromatic karyoplasm (examples pointed out by arrowheads). Histogram H depicts the number of *Adcyap1*-expressing peptidergic cells. Panel (**H**) illustrates the count of *Adcyap1* transcripts in peptidergic neurons. Histogram (**I**) indicates the number of *Adcyap1* mRNA transcripts in EWcp neurons. Graph (**J**) shows the count of peptidergic cells that contained *Adcyap1r1* transcripts. Histogram (**K**) depicts the count of *Adcyap1r1* transcripts in peptidergic EWcp cells. Bars in Figure (**L**) show the count of *Adcyap1* and *Adcyap1r1* co-expressing peptidergic EWcp cells. The number of PAC1R immunoreactive (ir) EWcp cells is shown in panel (**M**). The specific signal density (SSD) of PAC1R signal is shown in (**N**). Gray bars: oil- and saline-injected controls; red bars: rats treated with rotenone; pink bars: benserazide/levodopa (B/L)-treated parkinsonian rats. N = 6–8. * *p* < 0.05, ** *p* < 0.01, *** *p* < 0.001, according to Tukey’s post-hoc test. Bars: 20 µm.

As described above, rotenone treatment reduced the number of peptidergic cells in the EWcp (Figure 6G). We counted the peptidergic cells that expressed *Adcyap1* mRNA, *Adcyap1r1* mRNA or both. We observed that most of the peptidergic neurons expressed *Adcyap1* mRNA and their number was affected in the model (Figure 6H, ANOVA: F2,19 = 4.33; *p* = 0.028) as rotenone decreased their count (*p* = 0.01), while B/L treatment had no effect (*p* > 0.05). In line with this, the cells’ *Adcyap1* mRNA content (Figure 6I, ANOVA: F2,19 = 4.43; *p* = 0.026) decreased in parkinsonian rats (*p* = 0.012), regardless of B/L therapy (*p* > 0.05). Similarly, the count of *Adcyap1r1*-expressing cells (Figure 6J, ANOVA: F2,19 = 7.24; *p* = 0.004) was also reduced (*p* = 0.011) without a considerable effect of B/L (*p* > 0.05) and the magnitude of *Adcyap1r1* mRNA expression per cell also showed similar dynamics (Figure 6K, ANOVA: F2,19 = 6.69; *p* < 0.01) upon rotenone exposure (*p* = 0.023). The number of *Adcyap1* and *Adcyap1r1* co-expressing peptidergic EWcp cells (Figure 6L, ANOVA: F2,19 = 5.90; *p* = 0.01) showed a decrease in parkinsonian rats (*p* = 0.017), again regardless of B/L administration. We did not detect a significant change in the count of PAC1R immunoreactive EWcp cells (Figure 6M, ANOVA: F2,18 = 1.92; *p* = 0.17) and in their PAC1R SSD (Figure 6N, ANOVA: F2,18 = 1.40; *p* = 0.27).

#### 2.2.6. Insular Cortex

**Figure 7:**
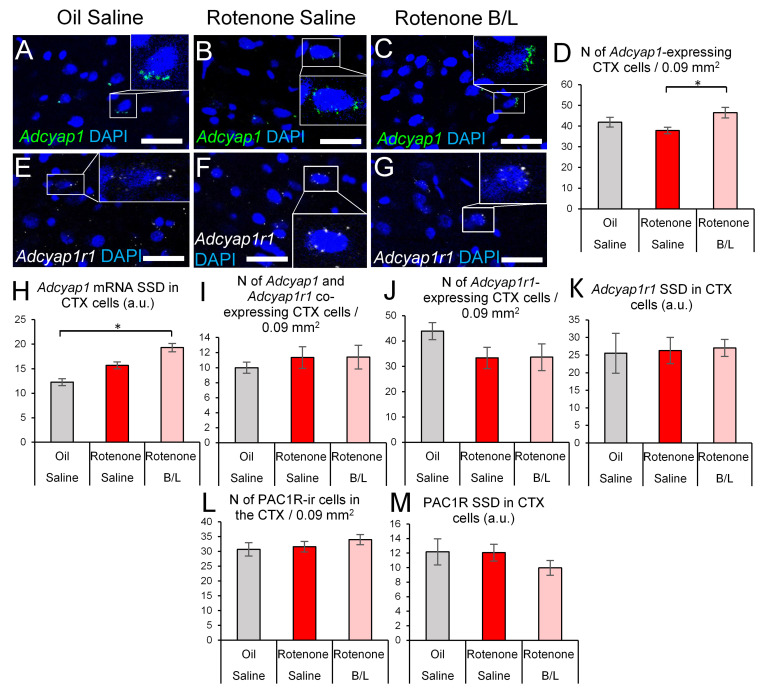
*Adcyap1* and *Adcyap1r1* mRNA expression in the insular cortex (CTX). Representative images show *Adcyap1* mRNA transcripts (green in (**A**–**C**), white arrowheads) and *Adcyap1r1* mRNA signal puncta (white, (**E**–**G**)) in sections counterstained with 4′,6-diamidino-2-phenylindole (DAPI, blue). Some cells were shown in higher magnification insets within the same panels. Histogram (**D**) shows the number of cortical cells that contained *Adcyap1* transcripts. The magnitude of *Adcyap1* mRNA specific signal density (SSD) is shown in (**H**). Panel (**I**) describes the count of *Adcyap1* and *Adcyap1r1* co-expressing cortical cells. Graph (**J**) shows the count of *Adcyap1r1*-expressing cells, while (**K**) illustrates the SSD of *Adcyap1r1* in these neurons. The count of PAC1R-immunoreactive (ir) cells is shown in (**L**), and the SSD of PAC1R immunoreactivity is shown in (**M**). Gray bars: oil- and saline-injected controls; red bars: rats treated with rotenone; pink bars: benserazide/levodopa (B/L)-treated parkinsonian rats. N = 6–8. * *p* < 0.05, according to Tukey’s post-hoc test. Bars: 20 µm.

Counting of *Adcyap1* mRNA-containing cells revealed the main effect of treatment as significant (ANOVA: F2,19 = 4.27; *p* = 0.29, Figure 7A–D); however, the Tukey’s post-hoc test did not detect significant differences between the oil-injected (control) and rotenone-treated rats (*p* = 0.26). In contrast, the B/L therapy was associated with higher number of *Adcyap1* mRNA-containing cells, compared to the rotenone-treated group; however, no difference was detected when compared with the controls (*p* = 0.19). Because we observed a confluent *Adcyap1* mRNA signal pattern in cortical neuronal somata, we measured the SSD of the signal (Figure 7A–C). Here we observed that the treatment significantly affected the amount of *Adcyap1* mRNA in the cells (ANOVA: F2,19 = 14.93; *p* < 0.001, Figure 7H). Rotenone injections tendentiously (*p* = 0.053) increased the *Adcyap1* expression (Figure 7H), which was further upregulated by B/L therapy compared both to the controls (*p* < 0.001) and to the rotenone-treated group (*p* = 0.009). The number of *Adcyap1* and *Adcyap1r1* co-expressing cortical cells remained unchanged in this area (ANOVA: F2,19 = 0.22; *p* = 0.79 Figure 7I).

The count of *Adcyap1r1*-containing cells (ANOVA: F2,19 = 1.24; *p* = 0.31, Figure 7J) and the amount of the *Adcyap1r1* mRNA in the cells (ANOVA: F2,19 = 0.03; *p* = 0.96, Figure 7K) was not affected in the CTX in the model. This was in full agreement with the unaltered cortical PAC1R immunoreactivity, as assessed by immunopositive cell counts (ANOVA: F2,19 = 0.78; *p* = 0.47, Figure 7L) and SSD measurements (ANOVA: F2,19 = 1.06; *p* = 0.36, Figure 7M).

## 3. Discussion

### 3.1. Behavioral and Morphological Findings Confirm the Validity of the Rotenone Model

In this study, the rotenone model of PD [44,45] was applied because it recapitulates the histological hallmarks of the disease in the rat, including dopaminergic cell loss and alpha-synuclein immunoreactive inclusions of the SNpc, with consequent reduction of dopaminergic fiber density in the CPu [17,44,45]. The present work, in line with other studies with rotenone [46], further supports the reliability of the model, as rats rendered parkinsonian suffered from serious deterioration of their motor skills in RPT. Our results also demonstrated the predictive value of this model, because B/L effectively improved the motor skills in accord with similar dopamine supplementation approaches [47,48]. Depressed mood and anxiety disorder are commonly associated with human PD (for a review see: [49]). In line with the present study, in our recent work [17], we observed that rats in the PD model also show these non-motor symptoms as exemplified by increased anhedonia and anxiety level in SPT and OFT, respectively. Importantly, the B/L therapy remained ineffective regarding these, suggesting that pharmacological intervention by improving dopaminergic neurotransmission does not have significant beneficial effect on the mood status. Nevertheless, the improvement of motor skills also contributes to the successful treatment of PD-associated mood disorders in human patients that requires medication targeting the monoaminergic systems [49]. At the same time, this also proves that mood disorders in PD cannot be explained by nigrostriatal dopaminergic neurodegeneration that the loss of motor skills is attributed to. Indeed, the recruitment of serotonergic, ventral tegmental area dopaminergic and noradrenergic systems has been proposed [50]. To the contrary, most recently we failed to confirm the damage of these centers in the rotenone model, but we observed the contribution of the peptidergic EWcp to non-motor symptoms of PD [17]. This is in full agreement with the current findings, that the peptidergic EWcp cell count decreases, but here we add that the neuronal loss in the EWcp is associated with reduced expression of *Adcyap1* and *Adcyap1r1*, suggesting a diminished neuroprotective state in this area that is in line with increased neuroinflammation [17].

### 3.2. Neuroanatomical Considerations

In this work we provide morphological data for the expression pattern of the PACAP mRNA (i.e., *Adcyap1*), but we do not show the presence of PACAP peptide per se at the protein level. We have to state this as a true limitation of our study, but despite efforts with multiple commercially available antibodies, we had to give up the goal to provide morphological data on the PACAP peptide. The reason is that the tested sera either did not give a recognizable signal in the rat brain, or they gave well-recognizable immunosignal when we pre-tested those on sections containing the bed nucleus of the stria terminalis (BNST) or hypothalamus of PACAP knockout mouse brain sections, suggesting their low sensitivity in the rat brain and/or their cross reactivity with other antigens. Nevertheless, the RNAscope ISH reliably allowed us to visualize the *Adcyap1* signal puncta in the examined brain areas. Earlier studies at the mRNA level did not detect considerable PACAP mRNA expression in the mouse CPu and GP [51], which was confirmed by studies using reporter mice [52]. In contrast, we identified *Adcyap1* transcripts in these areas; however, the expression was low, with fewer than five transcripts per cell. This was in agreement with images of the Allen Brain Atlas [53]. The discrepancy with earlier studies might be explained by the higher sensitivity of the RNAscope technique that allows even single molecule detection [54]. In line with this assumption, we and others detected stronger expression in the EWcp [18,53] and cortex [53] than in the striatum. The presence of *Adcyap1* mRNA in the EP and SNpc was in accord with earlier studies on wild type [53] and reporter mice [52].

As to the here observed distribution of *Adcyap1r1* and PAC1R, our findings are in accord with earlier neuroanatomical evidence in the rat, describing the occurrence of PACAP binding sites by autoradiography [55], *Adcyap1r1* mRNA distribution by ISH techniques [56,57] and immunohistochemical studies at the protein level [12]. It seems that the distribution of PACAP binding sites is evolutionarily conserved [10], as similar patterns have been shown both in rodents and in macaque monkeys [11,14]. This is also true for the here examined basal ganglia affected by Parkinson’s disease. In this study we focused on the PAC1R and we did not examine the receptors shared with VIP (i.e., VPAC1 and VPAC2) because earlier ligand displacement tests confirmed the significance of PAC1R in the neuroprotective effect of PACAP in monkeys [11] and rodents [7,58,59].

### 3.3. Dynamics of Adcyap1 and Adcyap1r1/PAC1R

Rotenone exposure decreased the count of both *Adcyap1* and *Adcyap1r1* mRNA-expressing cells in the CPu, GP, in the dopaminergic SNpc and in the EWcp, while no effect was observed in the EP and CTX. The reduction of these cell counts in the CPu does not mirror the result of neurodegeneration, because we did not observe reduced cell counts using the neuronal marker NeuN [17]. It is more likely that the downregulation of mRNA expression in viable cells explains these findings. Indeed, when we assessed the magnitude of *Adcyap1* mRNA expression in the cells, a similar pattern was observed, except that no decrease occurred in the GP. The semi quantitation of *Adcyap1r1* mRNA also revealed the downregulation in the same regions, but in contrast to the cell counts here, the *Adcyap1r1* mRNA content of the EP was also affected.

These findings suggest that the reduced *Adcyap1* and *Adcyap1r1* expression and presumably dampened PACAP/PAC1R signaling may contribute to the functional deficit of the CPu, because the reduction of PAC1R protein content upon rotenone treatment was significant here also. This is in agreement with studies on striatal cell lines and a genetically modified mouse model of Huntington chorea [60]. The B/L therapy reversed the decrease in *Adcyap1* mRNA cell count only in the CPu, suggesting that the improvement of motor skills may be at least in part explained by increased *Adcyap1* expression in the CPu. This recovery may have affected other, here not examined, messengers, such as brain derived neurotrophic factor (BDNF), which also interacts with PACAP/PAC1 [60]. Indeed, levodopa treatment was shown to increase the corticostriatal BDNF expression that helps to restore the compromised neuronal plasticity in the stratum in PD, which in turn improved motor skills, but may also contribute to the development of dyskinesias [61,62]. We saw earlier that the levodopa treatment improved the PAC1R immunoreactivity in the caudate nucleus of Parkinsonian monkeys in the MPTP model [14]. Although in that study we did not measure the *Adcyap1* expression, it seems that, in rats, the improvement requires the upregulation of the ligand expression, while that of the receptor plays a subordinate role in contrast to monkeys, where the PAC1R was upregulated by the therapy. On the other hand, this observation may be attributed to the differences between the two models we applied (i.e., MPTP vs. rotenone) and it is also important to mention that levodopa administration may reverse some, but not all, functional and morphological hallmarks of PD [63,64,65,66,67]. Further, the effect of rotenone in the EP was limited because only the count of *Adcyap1r1* transcripts per cell was reduced, but not the count of cells that contain *Adcyap1r1* transcripts. This differs from that which we observed in the globus pallidus internus, the corresponding area in monkeys, where the levodopa therapy improved the downregulated PAC1R content in parkinsonian macaques [14]. The comparison of the present findings in the SNpc with our earlier observations in monkeys reveals that, in rats, more than 80% of SNpc/TH neurons carry PAC1R; while in macaques, this was observed only in 3% of the dopaminergic neurons. This suggests that PACAP may have a stronger neuroprotective effect in the SNpc/TH neurons of rats than in monkeys. This notion is supported by our important finding that those dopaminergic SNpc cells that co-expressed both *Adcyap1* and *Adcyap1r1* (Figure 4J) were not affected by neurodegeneration in the rotenone model, while the TH cells that expressed only one of these mRNAs were susceptible for neurodegeneration (Figure 4G,K). This suggests the possibility of an autocrine neuroprotective PACAP/PAC1R signaling mechanism that protects a subpopulation of dopaminergic cells. The approval of this awaits further experimentation, but similar mechanisms were demonstrated in cerebellar granular [68], PC12 [69] and brain microvascular endothelial cells [70]. In line with this, a large body of evidence in multiple species suggests the cytoprotective action of PACAP in various toxic models of PD including 6-OHDA [25,32], salsolinol [26], MPTP [27,28,71] or rotenone [29,30,31,32]; for reviews see [8,23].

When studying our results, one may come to the idea that the rotenone exposure induced a brain-wide decrease in the mRNA expression. Our results do not support this, because a nearly significant (*p* = 0.053) elevation of *Adcyap1* mRNA expression occurred in the insular cortex that fits well with the observations of Broome et al. [31] in the prefrontal cortex. Similarly, the count of *Adcyap1* mRNA-expressing cells even increased in the neurons of SNpc that did not express TH, while the magnitude of expression was reduced in the dopaminergic neurons. The increased number of non-dopaminergic *Adcyap1*-expressing SNpc cells suggests a cell-type-specific compensation, by increasing the expression of PACAP. On the other hand, we did not observe a selective loss of SNpc cell populations that carry or lack PAC1R because their proportion did not change in the model, and a similar decrease was observed in the number of both dopaminergic and non-dopaminergic SNpc cells (Figure 4L,M). We assume this corresponds to a true cell loss because the lower cell counts cannot be explained by lower intensity PAC1R immunoreactivity, as PAC1R SSD values did not show significant changes. The counting of dopaminergic neurons also proved the cell loss in the SNpc, which further supported the validity of the model [17,31,44,45,72]. Indeed, systemic [23] intracerebroventricular [59] or intra-SNpc [32,33] PACAP administration has a neuroprotective effect via upregulation of its receptors [58,73]. In line with this, the role of PAC1R in neuroprotection was also shown [10,58,74], including pharmacological tests using maxadilan, a specific PAC1R agonist [75].

The above-discussed changes may have contributed to the alteration of motor skills in our rat model. As to the non-motor symptoms of PD including depression and anxiety, we recently showed that neuron loss of the EWcp also contributes to these psychopathologies in the rotenone model [17]. Because the peptidergic EWcp cells, besides multiple other neuropeptides, express both *Adcyap1* [18] and *Adcyap1r1* (this study) there is also a possibility of an autocrine neuroprotective PACAP/PAC1R signaling in the EWcp. Therefore, the downregulation of these mRNAs may be associated with the damage and disturbed functioning of these neurons. In line with this assumption, in our earlier studies on PACAP knockout and heterozygous mice, we saw the disturbed functional morphology and reduced adaptation capacity of peptidergic EWcp cells accompanied with a depression-like phenotype and increased anxiety [19,20,21,22,76,77]. On the other hand, beyond the EWcp, multiple mood control-related brain areas contain PACAP and PAC1 (e.g., hippocampus, BNST, amygdala) [51]. As these centers are affected in the late phases of PD [43], future tests will determine how PACAP/PAC1 signaling in these areas contribute to psychopathologies in advanced PD.

## 4. Materials and Methods

### 4.1. Animals

Male Wistar rats from the colony (original breeding pairs purchased from Animalab Kft., Vác, Hungary) of the Animal Facility of the Anatomy Department, University of Pécs were housed in two-per-cage groups. Rats were reared in an air-conditioned environment set to a constant temperature and humidity, in standard polycarbonate cages (40 × 25 × 20 cm). Ad libitum access to standard rodent chow and tap water was provided. In vivo experiments were conducted based on licenses (BA02/2000-49/2017 and BA02/2000-83/2022) issued by the National Food Chain Safety Office in Hungary upon approvals of Animal Welfare Committee of Pécs University, the National Scientific Ethical Committee on Animal Experimentation in Hungary.

### 4.2. Rotenone Treatment

Five-month-old rats were injected for six weeks daily with rotenone [(R8875-1G, Sigma, Budapest, Hungary) (n = 34; 1.5 mg/kg/day rotenone sc, in 20 µL/kg/day dimethyl-sulfoxide (Fisher Scientific, Loughborough, UK) and 1 mL/kg sunflower oil vehicle (8000-21-6, Molar Chemicals Kft., Halásztelek, Hungary)] vs. vehicle-injected controls (n = 8). Four rotenone-treated rats, due to humane endpoint, and six others, showing less than 20% SNpc dopaminergic neuron loss, were removed from the study [17,78].

### 4.3. Benserazide/Levodopa Therapy

Half of the rotenone-treated rats received two (at 7 a.m. and 5 p.m.) sc injections per day, consisting of benserazide-hydrochloride (10 mg/kg, BP685, Merck, Darmstadt, Germany) and levodopa (2.5 mg/kg, PHR1271, Merck) dissolved in 0.5 ml 0.9 M NaCl saline, for 21 days. The other half received only vehicle (saline) injections in the last 21 days of the six weeks rotenone treatment period. Control rats that did not receive rotenone but its vehicle (oil) and also sc saline injections in the last three weeks of the experiment. Behavioral test results (see below) obtained from 8–12 animals per group were included in the statistics. Six to eight brains per group were processed for histology.

### 4.4. Sucrose Preference Test

Depression belongs to the non-motor symptoms of PD. The SPT was applied to determine the anhedonia level in rotenone-treated rats that is characteristic of depression-like state. Three days before testing, two drinking bottles were offered to the rats filled either with 1 *w*/*v*% sucrose solution or tap water. The place of the bottles was reversed on the second day. On the morning of the test day (4 days before perfusion), both bottles were removed from the cage and a water deprivation was applied between 6 a.m. and 4 p.m. Then, the two bottles were offered again for individually caged animals for 3 h. The sucrose preference was calculated according to the widely used formula [79].

### 4.5. Open Field Test

Anxiety is a commonly diagnosed non-motor symptom of PD. Therefore, OFT was performed three days before perfusion in a brightly lit box (50 × 50 × 38 cm) with a black background. Five-minute-long video recordings were analyzed with SMART Junior Tracking software (version 1.0.07. PanLab, Barcelona, Spain) to assess the time spent in the periphery of the box, to reveal the anxiety level [80]. We also measured the distance traveled to assess the rats’ locomotor activity as an indicator of bradykinesia.

### 4.6. Rotarod Test

RPT was used to test the deterioration of motor skills. Animals were first pre-trained three times to run on the accelerating rotating axis. For testing, rats were placed on the axis of the device (47750, Ugo Basile, Gemonio, Italy) and the time interval rats spent on the rotating axis was registered two days before perfusion [17,72].

### 4.7. Fixation, Tissue Collection, Sectioning

Rats were anesthetized with intraperitoneal urethane injection (2.8 g/kg, Merck KGaA, Darmstadt, Germany). Animals were transcardially perfused with 50 mL 0.1M phosphate-buffered saline (PBS, pH 7.4) and subsequently with 250 mL 4% paraformaldehyde in Millonig’s buffer for 20 min. The brains were collected and post-fixed for two weeks in the same fixative. Thirty-µm-thick coronal sections were cut from the olfactory bulb to the ponto-medullary junction, using a vibratome (Leica Biosystems, Wetzlar, Germany). Six representative series of sections interspaced by 150 µm were collected in sterile PBS with 0.01% sodium azide. Sections were transferred into sterile RNase-free anti-freeze solution at 4 °C and, after they were submerged, they were stored at −20 °C until labeling [81,82]. Four representative sections of all brain areas were selected manually, based on the atlas of Paxinos and Watson [83], for each labeling. The following coronal plains were selected as defined by the distance from Bregma [in brackets] CPu: [−0.36 mm–(−0.84 mm)], insular cortex (CTX) and GP: [−0.48 mm–(−1.20 mm) ]; EP: [−1.91 mm–(−2.64 mm)]; SNpc: [−5.16 mm–(−6.24 mm)] and EWcp [−5.88 mm–(−6.72 mm)].

### 4.8. Double-Label Immunofluorescence for PAC1R and Tyrosine-Hydroxylase

Coronal sections containing the CTX, CPu, GP, EP, SNpc and EWcp were stained. After 4 × 15 min 0.1M PBS washes, sections were permeabilized by 0.5% Triton X-100 (Sigma-Aldrich Kft, Budapest, Hungary) in PBS and subsequently treated with 2% normal donkey serum (NDS, Jackson Immunoresearch, Suffolk, UK) diluted in PBS for 60 min. The specimens were incubated overnight at room temperature in the cocktail of the primary antisera: rabbit anti-PAC1R (1:500, Cat. No.: AVR-003, Alomone Laboratories, Jerusalem, Israel, lot # AN0102) and mouse monoclonal anti-tyrosine-hydroxylase (TH, 1:1000, Cat. No.: T2928 Sigma-Aldrich, Hungary) diluted in PBS containing 2% NDS. After 2 × 15 min washes in PBS, the sections were placed into a mixture of secondary antibodies containing cyanine 3 (Cy3)-conjugated donkey anti-rabbit (1:500; Cat. No: 711-165-152 Jackson Immunoresearch Europe, Ely, UK) and Alexa-488-labeled donkey anti-mouse (1:400, Cat. No.: 706-545-148 Jackson) sera for three hours in PBS. Then, after rinsing with PBS for 2 × 15 min, sections were mounted on gelatine-coated slides, air-dried and covered with glycerol–PBS (1:1) solution.

### 4.9. RNAscope In Situ Hybridization Combined with Immunofluorescence

Four sections of CPu, CTX, GP, EP, SNpc, and EWcp per animal were pretreated for RNAscope according to our recently developed protocol [84] optimized for 30-µm-thick PFA-fixed sections. Subsequent steps of the RNAscope protocol were performed according to the supplier’s (Advanced Cell Diagnostics, Newark, CA, USA) suggestions. The *Adcyap1* mRNA was visualized by fluorescein (FITC, 1:750) using a rat *Adcyap1* probe (Cat. No.: 400651) while the *Adcyap1r1* was detected with cyanine 5 (Cy5, 1:750) using a rat *Adcyap1r1* probe (Cat. No.: 466981-C3). Triplex positive control probes for rat tissue (Cat. No.: 320891) gave a well-recognizable signal in randomly selected sections of the areas of interest, while no signal was detected with triplex negative control probes (Cat. No.: 320871) upon hybridization and channel development (images not shown).

Subsequently, slides were further processed for immunofluorescence using rabbit anti-TH antibodies (1:2000, Cat. No.: Ab112, Abcam, Cambridge, UK), overnight at room temperature. After washes, Cy3-conjugated donkey anti-rabbit serum (1:500, Cat. No.: 711-165-152, Jackson) was used for 3 h. Sections were counterstained with 4′,6-diamidino-2-phenylindole (DAPI) and covered with ProLong Gold Antifade mounting medium (Thermo Fisher Scientific, Waltham, MA, USA).

### 4.10. Antibody Controls

Omission of primary or secondary antisera or their replacement by respective normal sera abolished the labeling in all cases (images not shown). The PAC1R antibody was raised in rabbit against a synthetic peptide (C)KVNRYFTMDFKHRH, corresponding to amino acid residues 474-487 of rat PAC1R. The specificity of PAC1R antibody (Cat. No.: AVR-003, RRID: AB_2756805, Alomone Laboratories) in the rat brain was tested by Western blot (https://www.alomone.com/p/anti-pac1-receptor/AVR-003 accessed on 21 July 2023) and by preabsorption to the corresponding blocking peptide (Cat. No.: BLP-VR003, Alomone Laboratories). The mouse monoclonal anti-tyrosine-hydroxylase antibody was generated against the N-terminal 40-152 amino-acid fragment of rat TH (1:1000, Cat. No.: T2928, RRID: AB_477569, Sigma-Aldrich, Hungary). The polyclonal rabbit anti-TH serum (Cat. No.: Ab112, RRID: AB_297840, Abcam, Cambridge, UK) was generated against the full-length TH protein isolated from rat pheochromocytoma. The sensitivity and specificity of our monoclonal mouse (http://www.sigmaaldrich.com/catalog/product/sigma/t2928, accessed on 21 July 2023) and polyclonal rabbit anti-TH antibody have been tested earlier in the rat brain by the supplier (https://www.abcam.com/tyrosine-hydroxylase-antibody-neuronal-marker-ab112.html, accessed on 21 July 2023) and in our laboratory [17].

### 4.11. Microscopy, Digitalization and Morphometry

Preparations were scanned by an Olympus FluoView 1000 confocal microscope (Olympus, Europa, Hamburg, Germany) equipped with a 40× (NA: 0.8) objective. Representative 300 µm × 300 µm areas were imaged in 1024 × 1024-pixel resolution with an optical thickness of 3.5 µm. The built-in settings of the FluoView software (Fv10-ASW; Version 0102) were used to excite (i.e., DAPI: 405 nm, FITC: 488 nm, Cy3: 550 nm, Cy5: 650 nm) and detect the fluorophores at their emission maximums in a sequential scanning mode. Blue (DAPI), red (Cy3), green (FITC) and white (Cy5) virtual colors were assigned to the dyes. For publication purposes, representative images were cropped, contrasted and edited using Adobe Photoshop software (version 7.0.1. Adobe, San Jose, CA, USA).

Morphometry, including counting of cells and signal dots of the RNA labeling, densitometry was performed on non-edited digital images by ImageJ software (version 1.52a, NIH), by a skilled colleague who was not informed about the identity of specimens. In cases when the fluorescent signal dots were well distinguishable, we counted the number of puncta per cell. Confluent signal puncta or diffuse cytoplasmic fluorescent signals were semi-quantified by measuring the cytoplasmic signal corrected for the background, yielding the specific signal density (SSD) [14,20]. Every quantitation was carried out on four images per animal and brain area. These four values were averaged and represented one animal in the statistical tests.

### 4.12. Statistics

Data were presented as mean of the group ± standard error of the mean. After confirming the normal distribution and homogeneity of variance of our datasets, one-way analysis of variance (ANOVA) followed by Tukey’s post-hoc test was performed. In a few cases a logarithmic data transformation was necessary to obtain a normal data distribution. Few outlier data beyond the 2-sigma range were excluded from the statistical assessment. Alpha was set to 5% in all cases.

## 5. Conclusions

In this study, we show that the rotenone treatment caused a marked decrease in the expression of *Adcyap1* and *Adycap1r1* mRNAs in the CPu, GP, SNpc and EWcp. The B/L therapy reversed the decrease in *Adcyap1* only in the CPu. This suggests that the reduced neuroprotective capacity via PACAP/PAC1R may contribute to the observed motor and non-motor symptoms. Therefore, future pharmacological interventions via PAC1R may help to improve the motor skills and mood status. Our current results, in comparison with our earlier studies in monkeys [14], further suggest the evolutionarily conserved neuroprotective role of PACAP/PAC1R signaling. Further preclinical research is required to study whether artificial stimulation of the autocrine PACAP/PAC1R signaling may have therapeutic significance in animal models. Later, clinical studies may be started to determine whether intervention in the non-symptomatic or early phase of human PD via PACAP/PAC1 may help to stop the neurodegeneration or at least delay progression.

## Data Availability

The data presented in this study are available on request from the corresponding author.
